# Atlanta Society of Medicine

**Published:** 1904-04

**Authors:** 


### *SOCIETY REPORTS.*

### ATLANTA SOCIETY OF MEDICINE.

March 3, 1904.

Dr. Wolff read a paper on Toxic Dermatoses (see page 6).

DISCUSSION OF DR. WOLFF'S PAPER.

*Dr. J. W. Duncan:* Dr. Wolff in his paper on chronic poisoning pursued a course different from what I had expected, and I am glad that he did, because he brought out in a very lucid manner the various exanthems resulting from chronic poisoning.

Destructive assimilation and elimination are just as important as constructive assimilation, and if the two are equal, we are not in much danger of autointoxication or chronic poisoning. We eat too much meat for people living in a Southern climate. Meat diet gives too much work for the liver and kidneys, and this is one cause of so much kidney and liver trouble. We should eat less meat, and be more careful about what we put into our stomachs.

The essayist did not say anything about alcoholic poisoning. Too much whisky is likely to have a deleterious effect on the liver, and whatever interferes with the functions of the liver should be used with caution, as the liver as well as the kidneys is an important eliminator.

The excessive smoking of cigarettes is likely to cause the tobacco heart, if the smoke from the paper wrappers is inhaled, which is usually done. We should use our influence with boys to prevent the habit before they become seriously poisoned.

*Dr. Hubbard:* I have enjoyed listening to Dr. Wolff's paper on the cutaneous manifestations of chronic poisoning, as he confines himself largely to that phase of the subject.

His paper was elaborate and showed careful study and preparation ; but, as I am not familiar, to any great extent, with skin diseases in any form, I desire to discuss the subject largely from the standpoint of autointoxication. This to me is one of the most interesting subjects in our profession to-day, and should command a great deal more study and scientific research by original investigators than it has in the past.

I think the medical profession has largely gone daft in their search for germs as a causative factor in the production of pathological conditions. If they would cease this illusive chase after the invisible, and devote more time to the study of poisons produced by the human organism itself, I think they will sooner arrive at a rational therapeutics and a more thorough understanding of diseases.

It seems to me that the bacteriologist has practically reached the limit and that the organic chemist must step in and supply the missing link between an established bacteriologic science and a rational therapeutics. The bacteriologist tells us that it is not the germ, but the toxin it produces, that causes the symptoms, but he can not tell us the chemical composition of that toxin; he can not give its physiological or pathological effect, except from a clinical standpoint, nor can he supply the chemical antidote to render it harmless.

Investigations along this line should not be confined to poisons produced by germs, but rather to the products of tissue metamorphosis destructive, which is constantly going on in the human organism.

When we remember that man forms in his liver enough poison in eight hours to kill him, and the urine eliminates but one-half, we have some insight into the toxicity of our own products. (Park.)

The well-known fact that total suppression of urine in a short time will result in death is a glaring illustration of the toxicity of this excretion. When we add to that the fact that if the skin is covered with some impervious material, death would also result, it needs no argument to convince any intelligent man of the importance of keeping the emunctory organs in a healthy and active condition. In other words, to keep up nutrition to the proper height and stimulate excretion; to eliminate the toxic products of tissue metamorphosis destructive should be the aim of the intelligent practitioner, both in health and disease.

The proper maintenance of this balance may be considered an explanation of that vague term in individuals called "constitution."

How many men, perhaps, have gone to early graves because when acquiring some acute disease they were suffering with a torpid liver, constantly accumulating poison therefrom, as well as deficient secretion of urine and constant accumulation of urea in the system.

This is why the surgeon of to-day so thoroughly prepares his patient for an operation by seeing that the liver is active, the bowels thoroughly purged and the kidneys secreting well; and the experience of all surgeons demonstrates the correctness of this treatment.

Now as to why man's organs do not functuate properly all the time and maintain this balance between the absorption of nutritive material and excretion of waste products is the problem for the physicians to solve. Proper exercise, we all know, is an important thing in maintaining this balance, but I will not enter into the therapeutics of autointoxication.

I am of the opinion that a large number of our chronic diseases of the liver, kidneys, and perhaps joint structures in the form of chronic rheumatism, are due primarily to an accumulation of these toxins, which perhaps has been going on in the system for years.

Who can gainsay that Bright's disease may not be the result of an attempt on the part of the kidneys to excrete products which the stomach and digestive apparatus has not properly prepared for elimination. And it has been thoroughly demonstrated that the liver acts as a sentinel, by the fact that the blood of the portal vein, loaded with poisonous products absorbed from the alimentary canal, is a great deal more poisonous than the blood after going through the liver, showing conclusively by this investigation, as well as by the toxicity of the bile when given to animals, that the liver plays an important role, perhaps in prevention and causation of diseases.

The alimentary canal, especially the lower part, was intended by nature as a sewer to drain from the system these toxins and other obnoxious products, and every practioner is well aware of the result of chronic constipation in his patients, and chronic constipation nearly always mean an inactive liver, at least in part; and when the liver does not perform its function properly, urea is not produced in sufficient quantity, and consequently, the natural stimulus to the secretion of urine is lessened, and we have a diminished amount.

How often do we meet in our daily rounds constipated people (women more especially), with headaches, neurasthenic symptoms, and the general explanation that they "feel bad."

The quickness with which purgatives and diuretics for a few days will make the worst of these symptoms pass away demonstrates their causation and the importance of elimination, another instance where therapeutic results furnish the basis for pathological investigation.

Dr. R. R. Kime read a paper on the Imperfect Development of the Uterus. (See page 1.)

DISCUSSION OF DR. KIME'S PAPER.

*Dr. Davis:* Dr. Kime is to be congratulated on the favorable results obtained after the manner of treatment outlined. I regret that he did not enter a little more into the embryogenesis of the uterus, as it is impossible to understand the failure of development without remembering a few essentials in the development of the uterus. Remember that fusion of Müllerian ducts takes place between the eighth and twelfth week of fetal life and the septum disappears in the lower two thirds. Now, an arrest of development prior to this would result in a mere rudiment of a uterus and during this time in a duplicity of the organ, uterus duplexus, uterus unicornis, etc.

Now, from twelfth to twentieth week the uterine cornua are distinct; arrest at this time leaves a uterus bicornis, uterus bifida, etc.

From twentieth month to sixth year fundus enlarges and becomes more proportioned to cervix, the folds begin to disappear from body of uterus, making distinction between fetal and infantile uterus. We can see from this that in certain cases there may be an absence of uterus and a full development of ovaries. In such cases the plan outlined by the speaker would be useless and only the removal of the ovaries would be of any benefit.

The plan outlined is not entirely devoid of danger and should not be adopted without reasonable hope of success. Most gynecologists can not report such gratifying results, and the method is as yet open to serious debate.

MARCH 17, 1904.

Dr. Stephen Barnett read a paper on Displacements of the Uterus.

DISCUSSION OF DR. BARNETT'S PAPER.

*Dr. Earnest:* After congratulating Dr. Barnett for giving us a very practical paper upon a subject of great interest on account of its frequency and the marked influence upon the health and happiness of our patients, I wish to say: In the first place, I believe that nearly all displacements are pathological, and should be so regarded, and that retrodisplacements, as a rule, should be corrected. Now and then you find a retroversion either with or without adhesions that has been known to exist for a long time without producing symptoms. Such cases should be let alone. If there is such a thing as a recent retroversion that produces no symptoms, I hold that it should be corrected without waiting for trouble to be set up. Many of these recent cases can be cured without surgical intervention. In certain quarters the pessary at one time, and even now, has been entirely discarded. I have never given it up, but have always found a limited field for its use. As suggested by Dr. Kime, the proper adjustment and management of pessaries necessarily requires a reasonable amount of mechanical genius. I wish to enter my unqualified protest against the once popular treatment of retrodisplacements by tamponing the vagina. Such a procedure gradually dilates and thins the vaginal walls and causes the absorption of neighboring tissues so that soon all support from the floor of the vagina is destroyed and the parts are left in a condition that makes the use of a pessary impossible. The lifting up of a retroverted uterus by a properly fitted pessary is sometimes all that is necessary to effect a cure. When a uterus has been held in position for some time a smaller pessary may be substituted, and in favorable cases all support gradually withdrawn.

Of course, we recognize the fact that a large per cent. of serious displacements can only be cured by surgical means, but we should not forget that now and then there is a case for treatment by pessaries, hot water and general medication.

For several years I have made it a point not to dismiss a case of confinement until I knew the uterus was in place.

*Dr. R. R. Kime:* It is refreshing in this day of surgical tendencies to hear such a paper read as this.

I indorse the views set forth in the paper. In days past pessaries were extensively used; of late they have been discarded by the surgeon, yet they have a legitimate useful field. In days past the perineum was supposed to support the uterus, but now we recognize, as the essayist states, that the supports are more from above and suspend the uterus rather than support from below.

We also condemn the use of the sound in replacing the uterus; it should be done by manipulation—with the fingers if necessary; put patient in knee-chest position.

While in that position the two fingers of the right hand may be used to advantage by pressing up behind uterus in posterior vaginal culdesac, making transverse movements with the fingers, but not too heavy pressure, thus carrying fundus up; then bring finger in front of cervix, carrying it backward and tilting fundus forward.

The use of Sims's speculum in this position, ballooning vagina, then pressure with a pledget of cotton held in forceps, as stated, is efficient.

A pessary should be adjusted only in suitable cases; not in a slipshod, haphazard way, but accurately fitted to each individual case, recognizing existing conditions. To properly adjust a pessary in many cases requires mechanical skill.

The displacement should be corrected, and many cases will require preparatory treatment before the proper pessary can be used.

We have a class of physicians or gynecologists that claim the position of the womb is of very little importance, so long as the womb itself and adnexa are not diseased.

While we recognize that the uterus is not a fixed organ and has normal movements within certain range, yet we feel there are certain displacements that are abnormal.

While the displacement is not a disease of itself, it favors the development of disease and aggravates diseased conditions when present.

We have displacement of the stomach, intestines, kidney, etc., and many recognize that such conditions often interfere with the normal functions of such displaced organs; in some produces nervous disturbances; in others tend to produce diseased conditions.

The uterus having a more complicated, nervous, lymphatic and vascular supply, certainly would be as likely, if not more so, to produce disturbance from displacements.

If displacements amount to nothing, then an immense amount of operative work has been done that is needless and uncalled for.

Gynecologists have seemed to vie with each other in developing operative measures for displacements of the uterus.

We have the vaginal shortening of the round ligaments, Pryor's culdesac operation; various extra- and intra-peritoneal shortening of the round ligaments, operations on the broad ligaments, utero-sacral ligaments, hysteropexia and suspension of the uterus. Why all this fuss if displacements amount to nothing?

As to prophylaxis, there is one point to which I desire to call especial attention, as I feel it has done more harm and has been more productive of displacements of the uterus than any one thing.

It is the keeping of the parturient woman on her back for ten or twelve days after delivery.

The womb is large and heavy, the ligaments have not regained their normal tone and strength, as they all, unless it be the utero-sacral ligaments, participate in the hyperplasia of pregnancy; and hence, not in a condition to hold the womb forward when in a dorsal position.

If evolution be true we should try to imitate nature by keeping the patient in exaggerated Sims's position several hours each day, changing from side to side; also, have patient, unless some special contraindications, get up over vessel for bowels and kidneys to act so as to favor drainage.

In many cases where retroversion exists, and even other diseased conditions, by appropriate treatment after labor and abortion they can be corrected without surgical measures. As soon after delivery as the danger of infection is over the womb should be carried forward; if necessary, with patient in Sims's position; if it will not remain so, a wool support (antiseptic) is placed behind fundus so as to support it until a hard-rubber pessary can be safely adjusted.

(At this time copious hot vaginal douches are essential; boric acid and alum add to their efficiency.)

I never use tampons in these cases, but a small, soft, spongy, wool support behind fundus; one that will not distend vagina and cause relaxation. Where retroversion, subinvolution, slight adhesions, etc., exist previous to pregnancy, this line of treatment following parturition will relieve many cases.

